# Factors affecting HBV vaccination in a Medical training College in Kenya: A mixed methods Study

**DOI:** 10.1186/s12889-020-8158-2

**Published:** 2020-01-13

**Authors:** Anne Njeri Maina, Leah Chebet Bii

**Affiliations:** 1 Department of Medical Microbiology, University of Nairobi, P.O Box 19676-00202, Nairobi, Kenya; 2 Kenya Medical Training College, Nairobi, P. O BOX, Nairobi, 30195-00100 Kenya

**Keywords:** HBV vaccination, Vaccine uptake, Health care Students, Kenya

## Abstract

**Background:**

Hepatitis B Virus (HBV) is highly endemic in Sub-Saharan Africa with 70 to 90% of the population becoming infected before the age of 40 years. Healthcare workers (HCWs) including healthcare students (HCSs) are at an increased risk of contracting HBV due to occupational exposure. HCSs are especially at a high risk because of their inexperience with infection control procedures and insufficient knowledge about the level of risk when dealing with patients. Despite the availability of an effective vaccine, and its recommendation by Kenya’s Ministry of Health, few HCW and students are vaccinated. The aim of this study was to evaluate the influence of awareness, attitude, practices, and access factors on hepatitis B vaccination uptake by HCSs at Kenya Medical Training College (KMTC).

**Methods:**

This was a concurrent mixed methods study. For the quantitative arm, a structured questionnaire was used to assess the awareness, knowledge, attitudes and practices towards HBV disease and vaccination. Accessibility of the HBV vaccine in the participating campuses was also assessed. Two FGDs were carried out: one comprised of student representatives of the participating campuses while the second comprised of members of staff. Quantitative data was analysed using STATA (version 15) while NVIVO (version 11) was used for qualitative data.

**Results:**

Out of 634 students invited to participate in the study, 487 participated (response rate 76.8%). Majority of the respondents were from Nairobi Campus (44.2%) and from the Department of Nursing (31.2%). HBV vaccine uptake rate was 85.8% while the non-vaccination rate was 14.3%. Full vaccination was reported by only 20.2% of respondents. The major reason for not receiving the recommended doses was the unavailability of the vaccine when students went for it. The qualitative study revealed challenges in the implementation of the vaccination program at KMTC.

**Conclusions:**

Full vaccination rates remained low despite good knowledge of HBV infection and positive attitude towards vaccination. There is therefore need to streamline vaccination programs in medical colleges to ensure availability and accessibility of the vaccine to healthcare students.

## Background

Hepatitis B virus (HBV) is one of the two major causes of chronic hepatitis, a precursor to liver cirrhosis and hepatocellular carcinoma [1]. In 2015, 257 million people were living with chronic HBV infection. More seriously, 1.34 million deaths occurred from viral hepatitis, higher than those from HIV [1]. This trend is expected to increase with time if the strategies outlined for elimination of hepatitis epidemics as a major public health threat by 2030 are not implemented. Briefly, these interventions include immunization; timely hepatitis B birth dose (HB-BD) for prevention of mother to child transmission; infection, blood and surgical safety in healthcare settings; harm reduction in people who inject drugs (PWID) and effective treatment for hepatitis [2]. Kenya is classified as highly endemic for HBV [3–10] with one study reporting a prevalence of 4.5% among health care workers (HCWs) [3]. Measures to combat the disease are therefore desperately needed [2].

According to the world health organisation (WHO), immunization against HBV is effective and safe and is one of the core synergistic interventions identified for its elimination. In particular, timely vaccination of children less than 5 years, and the introduction of the HBV birth dose (HBV-BD) have been singled out as being critical in eliminating perinatal transmission, which carries the highest risk of progression to chronicity [11].

In 2017, Kenya achieved an average coverage of 82% for the 3rd dose HBV childhood vaccination [12], falling short of WHO’s recommended coverage of 90%. Moreover, it has not yet introduced the HBV-BD despite evidence of prevention against chronic hepatitis﻿ citing few perinatal transmission rates. For HCWs, the monovalent HB vaccine is recommended in three doses at 0, 4 and 6 months [13].

Key to the realization of the goal of mitigating the effect of HBV on public health systems are HCWs. Their knowledge on HBV, attitudes towards immunization and practices are likely to determine the success, or lack thereof, of the interventions [14]. The impartation of these values can be maximized during training of health care students (HCSs).

During practical placements, HCSs immerse themselves in medical procedures with enthusiasm. This, combined with varying standards of supervision, may place them at risk of blood-borne infections of which HBV is a major concern. HCSs are especially at a high risk because of their inexperience with procedural skills, infection control procedures and also because they may have insufficient knowledge about the level of risk when dealing with patients [13]. The CDC recommends that due to this risk of occupational exposure to HBV, HCSs should receive vaccination before exposure to blood and blood products [14]. However, there is no explicit recommendation about the timing of vaccination of HCSs in Kenya.

Despite this recommendation, vaccination against HBV among HCSs in various African countries continues to fall below the target [15–18]. Some reasons for low vaccination coverage include transitory staff [19], busy schedule [20], lack of money to pay for the vaccine [21] and forgetfulness [22]. This is complicated by shortage of skilled HCWs, especially in low income countries, which may compel students to carry out procedures on their own especially in emergency situations [23, 24]. Unfortunately, the students are inexperienced and may have inadequate training in universal precautions [25, 26]. Furthermore, the migratory nature of the HCSs during their community oriented practical placements poses a serious challenge to the completion of a vaccination series once started. Enhancing the level of knowledge, perception of HBV vaccine safety and accessibility to the vaccine, would increase the proportion of HCSs vaccinated against HBV. This would in turn have a direct effect on the vaccination coverage of children, as vaccinated HCWs are more likely to recommend vaccination to others [27].

The aim of this cross-sectional mixed methods study was to investigate the awareness/knowledge, attitudes and practices of HCSs towards HBV vaccination. Further, we investigated the factors affecting the vaccine uptake of the HBV vaccine within KMTC’s campuses.

## Methods

We used a concurrent mixed methods study design. For the quantitative arm, a structured questionnaire was used to assess (i) the awareness/knowledge of students towards vaccination in general, and HBV vaccination in particular; (ii) their attitudes towards HBV vaccination in general, and the campuses’ participation in HBV vaccination of their students in particular; (iii) their practices involving HBV vaccination and prevention of infection; (iv) accessibility of the vaccine in the participating campuses. HBV vaccination status was determined by self-report.

For the qualitative arm, we conducted 2 focus group discussions (FGDs). The first FGD comprised 2 Student Representative Council (SRC) members from each participating college; 12 participants in total. The second FGD comprised 2 members of staff involved in the running of KMTC’s vaccine coordinating committee (VCC) from the participating campuses; 12 participants in total. Informed written consent was obtained from all the participants. The FGDs were carried out in English, which is the language of instruction in KMTC. Tape recorders were used to record the discussions and notes were taken as a backup for recordings.

### Study setting

The study was conducted at the Kenya Medical Training College (KMTC), the only public middle level health training institution under the Ministry of Health. Currently, KMTC has 65 campuses with a nationwide distribution with over 34,000 students, 8000 of whom graduate annually. It therefore contributes to 80% of Kenya’s health workforce. The study was conducted in June and July 2016 during the 2016/2017 academic year at which time there were 55 constituent campuses and a student and staff population of 21,209 and 1892 respectively. Out of the 55 campuses, 30 had an active vaccination program. This involved providing vaccination against typhoid fever and hepatitis B infections. The cost of the vaccination (35 USD) was included in the fees structure and new students were sensitized about it in the admission letters. However, the program was discontinued in September 2017, which occurred after this study had already been conducted.

### Sampling procedures

**Fig. 1:**
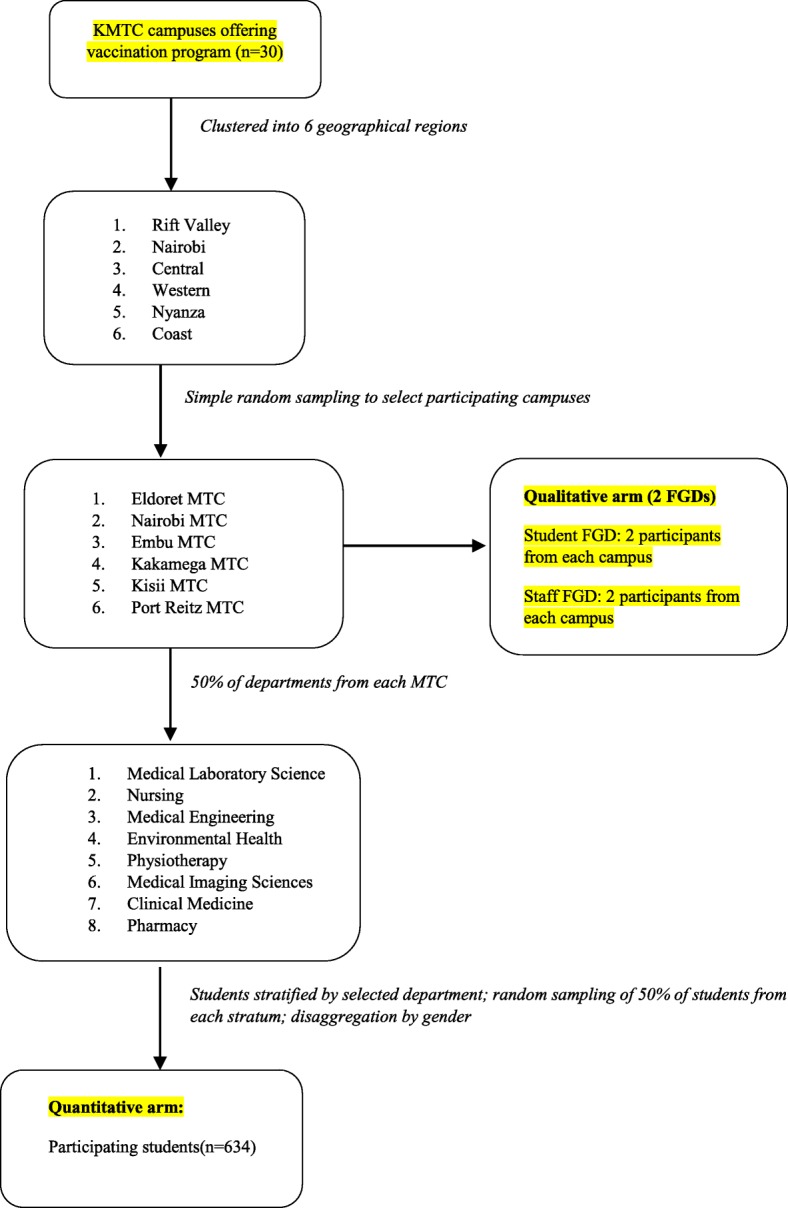
Flow chart showing participant recruitment for the quantitative study

For the quantitative study, a multistage sampling design was constructed to combine various sampling options (clustering, stratified, simple random and systematic sampling). Thirty [28] out of fifty five (55) campuses implementing the college vaccination program were included in the study. The 30 campuses were grouped into six clusters based on the geographical regions of the country. A simple random selection was used to pick a campus from each of the six clusters in the regions. Using random stratified sampling, 50% of the existing departments in each of the campuses were selected. A 50% representative sample of HCSs identified by their registration numbers was stratified according to the selected departments and year of study. The number of students obtained from each stratum was disaggregated by gender. The sample was determined using Systematic Random Sampling after the identification of the “starting point”. We moved from first year class through to third or fourth year students to find the study subject based on a determined interval for each campus (Fig. 1). Students who were attending short courses (less than 6 months’ duration) and those who were away on rural attachment or night off were excluded from the study.

For the qualitative arm, we conducted 2 focus group discussions (FGDs). The participants were purposively selected from the Vaccine Coordinating Committee (VCC) members of each of the 6 sampled campuses. Each campus VCC, chaired by the respective Deputy Principal in charge of academics, was responsible for the overall implementation of the vaccination program. A campus VCC was composed of two [2] members of the Student Representative Council (SRC) specifically, the Chairperson and the Health Commissioner, and six staff members. One FGD consisted of two SRC members from each of the 6 campuses. The second FGD consisted of 12 staff members composed of two VCC staff members selected by each of the six campus Principals.

### Sample size estimation for the quantitative study

The sample size was calculated using previously used methods [29]. We estimated a prevalence rate of vaccination of 50% due to lack of precise documentation of current KMTC rates. As a multistage sampling design, the sample size was calculated at 80% power with a 95% confidence level. Macfarlane et al (1997) asserts that in most immunization coverage cluster surveys, a design effect (DEFF) of approximately two [2] is usually acceptable for a multistage sampling design [28]. This study used a smaller DEFF equal to 1.5. Thus, the sample size calculation to assess hepatitis B vaccination coverage, assuming *p* = 0.5, *d* = 0.05 and *DEFF* = 1.5 was as follows:

$$ \frac{n={1.96}^2\times 0.5\times 0.5\ (1.5)}{0.05^2}=576 $$

10% was added to the computed number to give a final sample size of 634 (Fig. 1).

### Statistical analyses

#### Quantitative data analysis

Data was entered into MS Excel, cleaned and analyzed using STATA (version 15). Continuous data was reported using median/means and range. Categorical data was summarised as frequencies and proportions. Our primary outcome variable was vaccination status. We also sought to find out how many students had been fully vaccinated. We defined full vaccination status as having received 3 or 4 doses of the HBV vaccine; partial vaccination as having received 1 or 2 doses and no vaccination as having received no dose of the vaccine.

Cross tabulations with Pearson’s chi square were performed on several independent variables to assess the strength of associations with the vaccination status. Comparisons of individuals with full (3 or 4 doses) to those with partial (1 or 2 doses) and zero doses was done. Statistical significance was set at *p* values of less than 0.05.

#### Qualitative data analysis

The focus group discussions were transcribed and imported into NVivo (version 11). The transcripts were analysed using both pre-existing and emergent themes. Verbatim quotations of frequently expressed opinions were selected from the transcripts to illustrate the opinions of the staff and students.

#### Ethical approval

The study was approved by the Kenyatta National Hospital/University of Nairobi Ethics Research Committee (KNH/UoN ERC); approval number P725/11/2015. Permission was also obtained from KMTC’s Ethical Research Committee and its Administration.

## Results

A total of 487 students participated in the quantitative part of the study (response rate 77%). Female students were slightly more (50.8%) than male students (49.2%). Students from Nairobi MTC comprised 44.2% of participants while students from the Department of Nursing formed the majority (31.2%) of the participants. Most students were in their 3rd year of study (42.2%) and had been in the college for 3 years (39.1%) (Table 1).

**Table 1:** Demographic characteristics of the quantitative survey respondents

Factors	Characteristic	Frequency	Percentage(%)
Age (*n* = 449)	Mean (Std.Dev)	22.5 (2.81)	
	Median	22	
	Range	18,40	
Sex (*n* = 486)	Male	239	49.2
	Female	247	50.8
Campus (*n* = 486)	Kisii	53	10.9
	Kakamega	57	11.7
	Nairobi	215	44.2
	Embu	32	6.6
	Eldoret	60	12.4
	Portreiz	69	14.2
Department (*n* = 474)	Medical Laboratory Sciences	83	17.5
	Nursing	148	31.2
	Biomedical Engineering	50	10.6
	Environmental Health	32	6.8
	Physiotherapy	20	4.2
	Medical Imaging Services	26	5.5
	Clinical Medicine	58	12.2
	Pharmacy	57	12.0
Year of Study (*n* = 486)	1st year	118	24.3
	2nd year	158	32.5
	3rd year	205	42.2
	4th year	5	1.0
Duration at KMTC (*n* = 486)	< 1 year	106	21.8
	1 year	71	14.6
	2 years	106	21.8
	3 years	190	39.1
	> = 4 years	13	2.7

### Awareness and knowledge about Hepatitis B infection

**Fig. 2:**
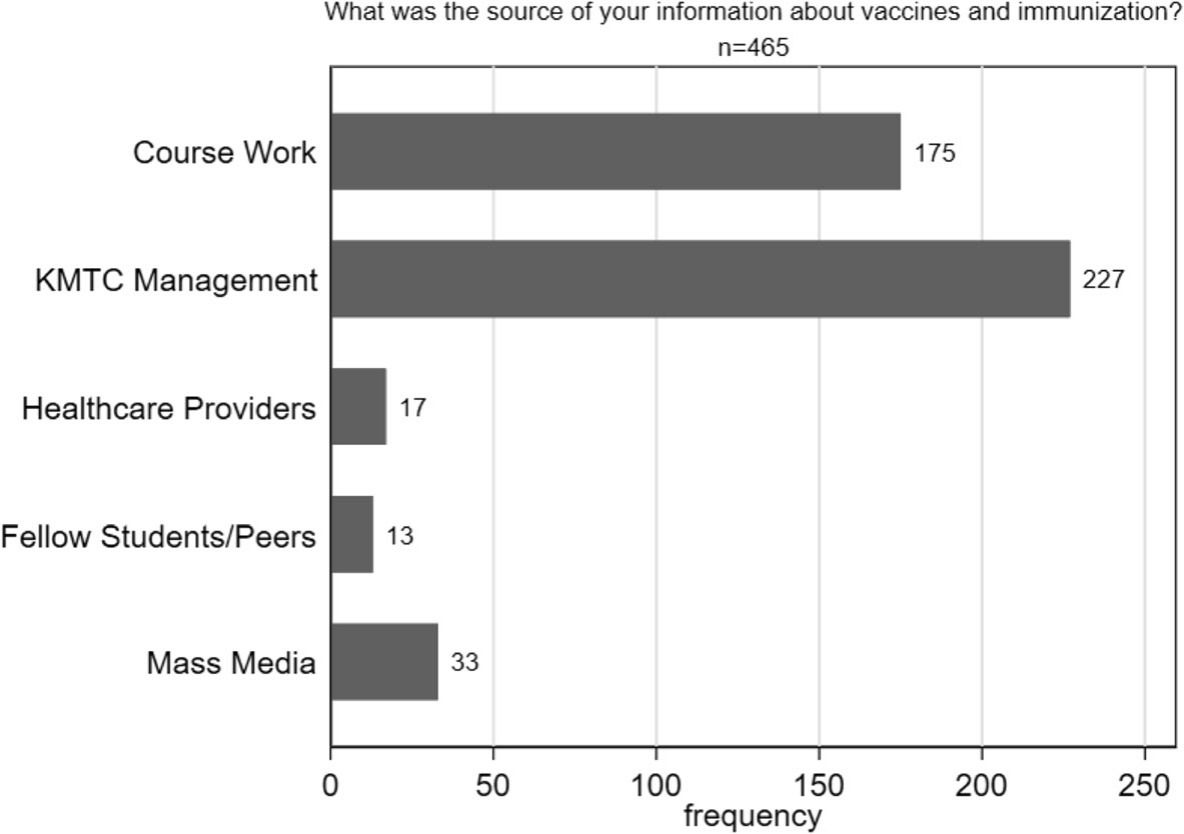
Bar graph showing reported most important sources of information about vaccination across all study sites

The reported major sources of information about vaccines and immunization were KMTC management and course work (Fig. 2). While the majority of students (94.6%) were aware that HBV vaccination was provided by the College’s vaccination program, fewer (53.3%) knew that vaccination against typhoid fever was also available. On the question of infectivity of HBV, 58.6% were aware that HBV is more infectious than HIV and that it can lead to development of liver cancer (59.5%). We also sought to find out the students’ knowledge on the known modes of HBV transmission as outlined by the WHO [30] (Table 2). Majority of them (76.8%) knew that HBV can be transmitted through contact with open wounds and cuts and transfusion of contaminated blood or blood products (88.1%) among others (Table 2).

**Table 2:** Student responses to the known modes of transmission of HBV

Which of the following are the modes of transmission of HBV?^a^	Yes *n(%)*	No n(%)
Transfusion of contaminated blood or blood products (*n* = 451)	429 (95.1)	22 (4.9)
Unprotected sexual intercourse (*n* = 430)	310 (72.1)	120 (27.9)
Mother to child transmission (*n* = 407)	301 (75.0)	106 (26.0)
Scarification, tattooing and shaving (*n* = 381)	226 (59.3)	155 (40.7)
Handling contaminated surfaces (*n* = 397)	271 (68.3)	126 (31.7)
Handling contaminated equipment (*n* = 401)	294 (73.3)	107 (26.7)
Splashes from contaminated fluids (*n* = 398)	334 (83.9)	64 (16.1)
Needle-stick injuries (*n* = 423)	392 (92.7)	31(7.3)
Cosmetic procedures (*n* = 395)	249 (63.0)	146 (37.0)
Dental procedures (*n* = 377)	223 (59.2)	154 (40.9)
Injecting drug use (*n* = 417)	372 (89.2)	45 (10.8)

^a^ All the modes listed are demonstrated modes of transmission of HBV [30]

### Awareness and knowledge about HBV vaccination

Most respondents (88.17%) believe that vaccination against HBV can protect one against acquiring the disease. A majority (75.3%) of respondents knew the correct mode of administration of the vaccine. However, only 43.2% knew that the hepatitis B vaccine is given in three doses. Majority of the students (73.0%) knew that individuals whose jobs involve contact with blood should be vaccinated against HBV (Table 3).

**Table 3:** Students’ responses to groups of people who should receive vaccination against HBV

According to the World Health Organization (WHO) recommendation, who should be vaccinated against hepatitis B virus?	*N*	%
Newborn babies (*n* = 374)	199	53.1
Children and adolescents who were not vaccinated in infancy(*n* = 482)	225	46.2
Individuals with multiple sexual partners (*n* = 482)	111	22.8
Individuals seeking treatment for Sexually Transmitted Infections or Human Immunodeficiency Virus infection (*n* = 482)	101	21.0
Injecting drug users (*n* = 481)	143	29.7
Individuals whose jobs involve contact with blood (*n* = 482)	352	73.0
Patients undergoing dialysis (*n* = 482)	123	25.5
Individuals with chronic liver disease (*n* = 479)	146	30.5

### Attitudes towards HBV vaccination

Most of the respondents (95.1%) felt that KMTC should be involved in hepatitis B vaccination of its students. Further, most students reported that they would recommend the vaccine to fellow students with the main reason for recommendation being to protect oneself from infection (Table 4).

**Table 4:** Students’ responses to whom they would recommend the vaccine and reasons for recommendation

Would you recommend the HBV vaccine for the following groups of people?	Yes (*n %*)	No (*n %*)
Fellow students (*n* = 452)	442 (97.8)	10 (2.2)
Newborns (*n* = 356)	233 (65.4)	123 (34.6)
Infants (*n* = 348)	234 (67.2)	114(32.8)
Adolescents (*n* = 393)	356 (90.6)	37(9.4)
Adults (*n* = 390)	345 (88.5)	45 (11.5)
Why would you recommend the HBV vaccine?		
To protect oneself (n = 449)	445 (99.1)	4 (0.9)
To protect patients (*n* = 378)	338 (89.4)	40 (10.6)
To protect your sexual partner (*n* = 357)	261 (73.1)	96 (26.9)
To protect others (n = 393)	361 (91.9)	32 (8.1)
To prevent mother-to-child transmission (*n* = 375)	301 (80.3)	74 (19.7)

**Fig. 3:**
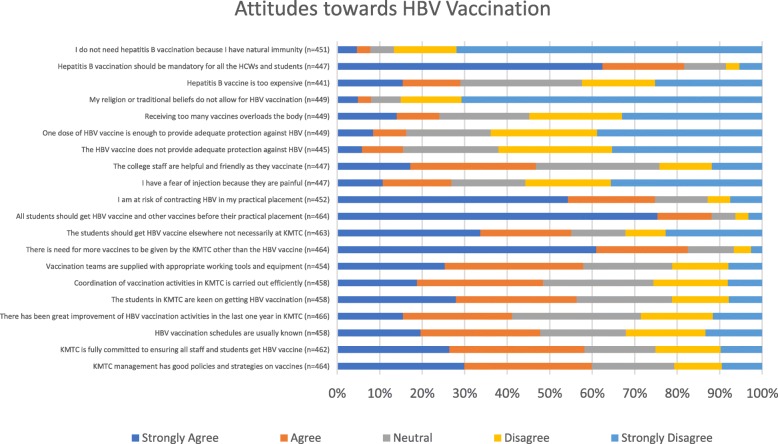
Students’ attitudes towards HBV vaccination and KMTC’s involvement in vaccination

Majority of students strongly agreed that all students should get vaccination against HBV before proceeding to their practical placement because of the risk of contracting HBV during clinical procedures. There was also strong agreement that HBV vaccination should be mandatory for all HCWs and students (Fig. 3).

### Practices

#### Vaccination against HBV

To investigate the students’ vaccination status, we asked the following questions: Have you ever been vaccinated against hepatitis B? Yes/No; If yes, how many doses have you received to date? There were 407 students who responded to both questions and these were classified into: Full vaccination(3 or 4 doses received); partial vaccination (1 or 2 doses received) and no vaccination (no doses received).

Majority of the respondents (349/407;85.8%) reported to ever having been vaccinated against HBV. However, full vaccination was reported by only 20.2% (82/407) with majority having received partial vaccination: 65.6% (267/407). No vaccination was reported by 14.3% (58/407) students. The main reasons for not having had full vaccination was that the vaccine was not available when they went for it (35.8%) and that the vaccine dose was not yet due (29.4%). Most students received the vaccine within their campuses (63.8% in the students/staff clinic; 31.0% within the college premises other than the clinic). In most cases, a college healthcare worker vaccinated the students (73.1%). There were no side effects reported by the majority of vaccinated students (67.8%) while swelling at the site of injection was the most reported side effect (57.6%).

#### Infection prevention during practical placement

During practical placement, 84.2% of students reported that they always put on gloves when carrying out clinical procedures such as cleaning wounds and cuts. However, 21.0% reported to having had a needle stick injury (NSI). Of those who reported NSIs, 18.3% took no action with only 38.7% reporting the matter immediately and getting post exposure prophylaxis that included the HBV vaccine.

#### Vaccine accessibility

Majority of students (85.3%) reported that the cost for HBV vaccination was included in the college fees. However, only 40.8% reported that the HBV vaccine is available in their colleges on a continuous basis, with 41.3% reporting that the schedule for each round of the HBV vaccination program is not well publicized. Despite this, 70.1% of respondents still prefer to receive the HBV vaccination within their campuses.

#### Association of vaccine uptake with sociodemographic characteristics

We investigated the association between HBV vaccine uptake and selected sociodemographic characteristics. We defined vaccination status as (i) No vaccination for zero doses received (ii) partial vaccination for 1 or 2 doses received and (iii) full vaccination for 3 or 4 doses received. The campus, year of study and length of time as a student had a statistically significant association with vaccine uptake (Table 5).

**Table 5:** Association of vaccine uptake with selected sociodemographic characteristics

	HBV Vaccination Status			Total	Pearson chi2	*p*-value
	None ^a^	Partial ^b^	Full ^c^			
Gender						
Male	30	122	43	195	*X*^*2*^ = 1.5380	*p* = 0.463
Female	28	145	39	212		
Total	58	349	82	407		
Campus						
Kisii	11	33	4	48	*X*^*2*^ = 34.4914	*p* = < 0.001
Kakamega	11	26	13	50		
Nairobi	18	120	37	175		
Embu	0	22	9	31		
Eldoret	10	21	15	46		
Portreitz	8	45	3	56		
Total	58	267	81	406		
Department						
Medical Laboratory Sciences	18	33	17	68	*X*^*2*^ = 55.4679	*p* = < 0.001
Nursing	13	90	19	122		
Biomedical Engineering	15	18	7	40		
Environmental Health	0	22	9	31		
Physiotherapy	0	14	4	18		
Imaging	4	9	7	20		
Clinical Medicine	1	46	8	55		
Pharmacy	5	30	8	43		
Total	56	262	79	397		
Year of Study						
1st year	31	46	6	83	*X*^*2*^ = 87.3760	*p* = < 0.001
2nd year	22	102	14	138		
3rd year	3	116	61	180		
4th year	2	2	1	5		
Total	58	266	82	406		
Length of time as student						
< 1 year	31	38	5	74	*X*^*2*^ = 115.7315	*p* = < 0.001
1 year	8	42	9	59		
2 years	9	74	12	95		
3 years	2	110	54	166		
> 4 years	8	2	2	12		
Total	58	266	82	406		

^a^ 0 vaccine doses received; ^b^ 1 or 2 vaccine doses received; ^c^ 3 or 4 vaccine doses received

#### Qualitative findings

The main themes that emerged from the two focus group discussions were (i) availability and accessibility of the HBV vaccines in the campuses; (ii) attitude towards the vaccination exercise; and (iii) institutionalization of the vaccination program. Quotes with frequently expressed sentiments have been added under each theme.

#### Availability and accessibility of HBV vaccine in the colleges

Participants in both FGDs felt that there was low availability and accessibility of the vaccine in the campuses. This was especially in those campuses far from KMTC’s headquarters (Nairobi). The main contributing factors cited were delays in supply of the vaccine, lack of transportation of the vaccines to the campuses, lack of vaccine storage facilities in some campuses, inadequate numbers of staff members to carry out the vaccination exercise and shortage of supplies needed for the vaccination exercise. Consequently, some students did not receive the full course of the vaccine prior to practical placement.

*“It has not been very easy, at times the vaccines are not there and no storage facility especially outside Nairobi and of course transportation [is a challenge]” [Staff 2].*

*“It [vaccine supply] is not [consistent] because some doses are missing and therefore, we are forced to give unrecommended doses. We have back logs and at times the students refuse to [go to] the wards.” [Staff 1].*

The shortage of staff members to carry out the vaccination exercise was expressed in both FGDs. This led to senior students working as vaccinators. The respondents felt that this had the potential to expose students to risks of receiving an injection from an unqualified practitioner.

*“Lack of qualified vaccinators [is a challenge]. In most colleges, we use senior students to help in the vaccination process”[Staff 1].*

“*… ..it [vaccination] is done by senior students who are not qualified …*.” *[Student 6].*

Poor timing of the HBV vaccine doses also arose in the discussions. Due to delays in supply of the vaccine, students were exposed to the practical attachment without having received the full course of the vaccine (3 or 4 doses).

*“I wish the supply [of the vaccine] can be consistent. We should get the vaccines before students go for their attachment … … .when the vaccine gets late, the students are already in the rural attachment and [it is] very difficult to get them.” [Staff 5].*

*“Yes [we should be vaccinated before practical attachment] because we are going to be exposed in the wards.” [Student 3].*

#### Attitude towards the vaccination process at KMTC

Students felt that the delay in getting the vaccine was due to an unconcerned administration. In addition, they felt that the administration had not put in place proper awareness creation channels about the availability of the vaccine to its students.

*“The college is not doing enough because there is no communication. They wait for the students to push for it …*” *[Student 9].*

Staff members felt that implementation of the vaccination program was not efficient. They felt that poor monitoring and assessment of the vaccine supply chain as well as lengthy procurement processes were to blame for delays.

“*… some institutions [campuses] only remember [to issue first dose to] new students and [are] not able to give the second dose. Major issue is procurement.”[Staff].*

#### Institutionalization and sustainability of the vaccination program

There were mixed feelings about the need to continue with the vaccination program at KMTC. While some staff and students felt that the program should continue albeit with some improvement, others felt that the program should be stopped and students allowed to get the vaccine outside the college.

*“The system can be improved … every campus should have its vaccine. Storage should first be improved.”[Staff 11].*

*“No, I don’t support the current structure but I would prefer it outside the KMTC.”[Student 9].*

#### Triangulation of quantitative and qualitative findings

Delay in receiving the vaccine was highlighted in both the qualitative and quantitative studies. The FGDs provided insight into the causes of the delays which included centralization of the vaccine procurement process in the headquarters thus necessitating transport of the vaccine to campuses, lack of transportation and storage facilities at the peripheral facilities. Inadequate staff members to carry out the vaccination exercise also affected the accessibility of the vaccine. While the quantitative study showed that majority of students supported the continuation of the vaccination program, the qualitative study offered insight into opinions both for its continuation and discontinuation.

## Discussion

In this study, we investigated the awareness/knowledge, practices and attitudes of healthcare students towards HBV vaccination at a middle level college in Kenya. We also investigated the accessibility of the vaccine in the participating campuses. Our study revealed that majority of the students were aware and knowledgeable about HBV infection and some of its various modes of transmission. However, we found that a lower number of students (43.2%) were aware of the recommended number of doses of the vaccine despite knowing the correct route of administration (75.3%).Our findings are similar to those among medical students in Cameroon that showed a majority had adequate knowledge of HBV infection and vaccine, and HBV transmission [31]. However, they are different from those found among medical students in India, Syria, Nigeria and Lao PDR, which found poor knowledge, and lack of awareness about hepatitis B, its routes of transmission, risk factors, and modes of prevention [32–35].

While a large majority of our respondents (85.8%) had ever been vaccinated against HBV, only 20.2% were fully vaccinated. A large number (65.7%) were partially vaccinated mostly due to absence of the vaccine from the campus clinics when students went for it. Our FGDs provided insight into the possible reasons for this. Low availability of the vaccine in most campuses was due to delays in receiving the vaccines from KMTC’s headquarters, lack of transportation and storage facilities in the campuses. Further, the awareness created about impending vaccination drives seemed not to be adequate. The rate for full vaccination among our respondents was lower than the rate reported among HCSs in Greece [30] but higher than that found in a study among medical students in Cameroon which showed a complete vaccination rate of 16.8% [31]. Non-vaccination in our study was reported by 14.3% of respondents which was lower than the 33.2% found among students in Uganda [36]. The existence of a vaccination program which encourages the uptake of the vaccine at KMTC could explain the lower non-vaccination rates.

Most respondents had a positive attitude towards vaccination against HBV due to its ability to protect an individual from getting the disease. Recommendations rates of the vaccine to various populations at risk of HBV acquisition however varied. Of the students surveyed, only 53.1% of HCSs would recommend HBV vaccination to newborns. While this is a slight majority, more HCSs need to know about the critical role of HBV-BD in the elimination of HBV. In addition, only 46.2% of the students reported that they would recommend catch-up vaccination for children and adolescents who did not receive the vaccine as infants. Given that catch-up vaccination of adolescents is important for a highly endemic country like Kenya, efforts should be made to inform HCSs on existing recommendations. Due to their critical future role in dissemination of knowledge and raising awareness among their communities, more educational efforts should be exerted on the students to enable them contribute to the prevention of HBV [35].

The respondents’ year of study and duration at KMTC were significantly associated with vaccine uptake. These findings are similar to those found among university students in Malaysia and Uganda [36, 37]. This is probably associated with the increased awareness of HBV by senior students as more coursework has been covered, as well as being in the college for a longer time, which would offer more opportunities for vaccination. Creating awareness about HBV vaccination among students in their early years could therefore increase vaccine uptake.

## Limitations

Despite providing insight into the vaccination status and associated factors among HCSs in Kenya, our study had limitations. One of the limitations was that only campuses that had the vaccination program in place at the time of the study were included. However, our multi-stage sampling method made sure that the participants were as representative of the student population as possible.

The vaccination status was based on self-report by the respondents and not by reviewing immunization registers. It is possible that the study design suffered recall bias. All participants were drawn from a middle-level public medical training institution, and hence may not be representative of medical students in universities and other HCSs in private training institutions. The qualitative arm of our study only had two FGDs. It is therefore difficult to make qualitative inferences. This study however offers a basis for future studies with larger sample sizes.

## Conclusions

Low full vaccine uptake was observed among the HCSs despite adequate awareness and knowledge of HBV disease and its modes of transmission; and positive attitude towards vaccination. The main reason for the low rate of coverage was unavailability of the vaccine when students went for subsequent doses. While having a vaccination program in place in a medical college is commendable, efforts should be put in place to ensure timely delivery and administration of vaccine doses.

## Data Availability

The datasets used and/or analysed during the current study are available from the corresponding author on reasonable request.

## Abbreviations

HBV: Hepatitis B Virus
HBV-BD: Hepatitis B vaccine birth dose
HCSs: Health care students
HCWs: Health care workers
SRC: Student Representatives’ Council
VCC: Vaccine Co-ordinating Committee
